# Do experts practice what they profess?

**DOI:** 10.1371/journal.pone.0190611

**Published:** 2018-01-05

**Authors:** Yun Zhou, Sudanthi Wijewickrema, Ioanna Ioannou, James Bailey, Gregor Kennedy, Debra Nestel, Stephen O’Leary

**Affiliations:** 1 Department of Surgery (Otolaryngology), University of Melbourne, Melbourne, Australia; 2 Department of Computing and Information Systems, University of Melbourne, Melbourne, Australia; 3 Melbourne Centre for the Study of Higher Education, University of Melbourne, Melbourne, Australia; 4 Department of Surgery (Austin), University of Melbourne, Melbourne, Australia; University of Westminster, UNITED KINGDOM

## Abstract

We investigated the variation of drilled regions of expert and trainee surgeons performing virtual temporal bone surgery to identify their compliance with standard drilling procedures. To this end, we recruited seven expert and six trainee ENT surgeons, who were asked to perform the surgical preparations for cochlear implantation on a virtual temporal bone. The temporal bone was divided into six regions using a semi-automated approach. The drilled area in each region was compared between groups using a sign test. Similarity within groups was calculated as a ratio of voxels (3D points) drilled by at least 75% of surgeons and at least 25% of surgeons. We observed a significant difference between groups when performing critical tasks such as exposing the facial nerve, opening the facial recess, and finding the round window. In these regions, experts’ practice is more similar to each other than that between trainees. Consistent with models of skills development, expertise and expert-performance, the outcome of the analysis shows that experts perform similarly in critical parts of the procedure, and do indeed practice what they profess.

## Introduction

The traditional apprenticeship model, where trainees practice and refine their surgical skills under the supervision of expert surgeons is regarded as the gold standard in ENT training [1, 2]. A shortage of cadavers, limited availability of experts, limitations in working hours of trainees, and lack of objective assessment methods has led to the development of new training methods such as Virtual Reality (VR) simulation. Surgical simulators typically provide the user with a 3D representation of the operative field, within which surgical tools are used to perform procedures on a “virtual patient”. Simulations may also render force-feedback and/or sound to improve the fidelity of the virtual operative experience. One of the advantages of using VR in surgical training is the opportunity to record performance metrics directly from the simulator, and from these, derive objective assessments of operative skill.

In our previous work, we have shown that expertise can be determined from simulator-generated metrics that relate to surgical technique [3, 4]. Here, we seek to test a dogma at the heart of surgical practice—that at critical stages of an operative procedure experienced surgeons will not deviate from a “standardized” technique. It may seem self-evident that this would be the case, but observation of expert surgeons in routine surgery show that they deviate from the didactic approach to the surgery that they teach. This in fact appears to be a reproducible characteristic of the expert; as a learner moves from foundational study through basic competence to true expertise, there is a move away from a protocol-driven technique to a flexible approach to a problem [5, 6]. This has been viewed as the essence of expertise [6, 7], and is thought to reflect an expert drawing upon a wealth of experience that can be adapted to each new situation. These observations are often in contrast to the way in which experts present their own approach to complex surgery—leaders in the field publish and present detailed protocols on how to deal with critical aspects of an operation. This begs the question as to whether experts actually do as they profess when dealing with the most crucial aspects of an operation. VR simulation provides a new way to test this, through the analysis of simulator-generated metrics.

The operative procedure evaluated in this paper is temporal bone surgery. The temporal bone houses the outer, middle and inner ears. It is dissected during operations to treat chronic otitis media, implant auditory prosthesis, treat diseases of the facial nerve or access the skull base [8, 9]. During these procedures, a surgical drill is used to remove the bone surrounding critical anatomical structures that are embedded within it, such as the facial nerve or the main venous sinuses draining the head. To investigate if and when expert surgeons develop their own “styles” of drilling in temporal bone surgery, we examine the variability of drilled areas in different regions of the temporal bone and relate this data to the perceived criticality of the corresponding phase of the surgery. We postulate that there will be less variability when accepted surgical opinion dictates that most caution needs to be exercised. The use of simulation in this way opens up for the first time an experimental method to study this question of whether experts do indeed follow standard guidelines they advocate when training mentees. These investigations could lead to a better understanding of how an expert surgeon behaves when there is perceived risk, enable better conceptualization of expertise, and in addition assist in the development of automated performance evaluation techniques.

## Materials and methods

### Simulation platform

**Fig 1:**
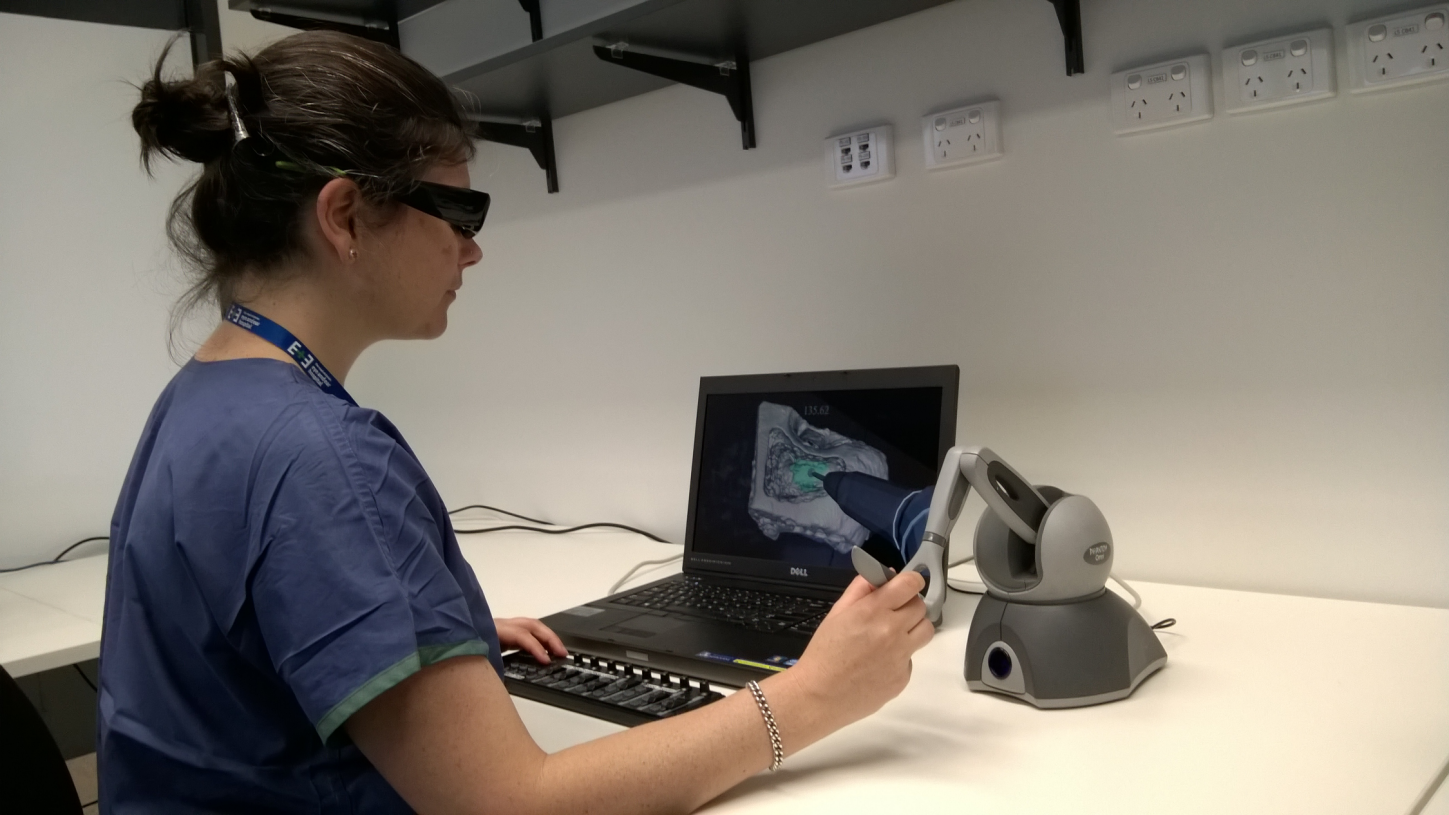
A surgeon performing temporal bone surgery on the University of Melbourne temporal bone surgery simulator. The image is presented on a 3D screen, with the user wearing 3D goggles. A haptic enabled 3D pointing device (right hand) is used to interact with the virtual model, and represented in the simulation as a drill (as shown on the screen). Simulation parameters are adjusted with a MIDI interface controller (left hand).

The surgical outcomes analyzed in this study were recorded on the University of Melbourne VR temporal bone surgery simulator [10]. In this simulator, major anatomical structures that need to be identified without injury during surgery, such as the sigmoid sinus, dura, facial nerve, chorda tympani, and round window membrane are represented in a 3D model of the temporal bone. Surgeons interact with the virtual temporal bone using a haptic device to drill the virtual temporal bone (see Fig 1).

### Experimental design

Participants undertook a specific operation of the temporal bone, involving the surgical preparation for cochlear implantation. In this operation, the bone of the mastoid is removed (“cortical mastoidectomy”), and then the bone between the facial nerve and the nerve for taste (the chorda tympani) is drilled away in order to enter the middle ear (to create a “posterior tympanotomy”). Finally, the surgeon enters the cochlea with a fine diamond burr (i.e. performs a “cochleostomy”). The cochleostomy provides the cochlear implant electrode access to the inner ear and is performed using the cochlea’s round window as a landmark to ensure that structures of the inner ear are not damaged. For this reason, the round window is exposed prior to undertaking the cochleostomy. In this operation, the critical steps are the performance of the posterior tympanotomy when the facial nerve and chorda tympani are at risk of injury, identification of the round window as this provides the surgical landmark for siting the cochleostomy, and then the actual placement of the cochleostomy.

Our hypothesis is that when experts perform this operation that there will be much less variation in the removal of bone during these critical steps of the operation than at earlier stages in the procedure. The hypothesis was tested by comparing the variance in bone removal for experts with that of surgeons who have had considerably less experience (trainee surgeons in Otolaryngology Head and Neck Surgery). Variance in bone removal was assessed for each of the steps of the procedure. We predicted that when the critical stages of the operation were reached (posterior tympanotomy, exposure of the round window, and cochleostomy) that there would be significantly less variance in the bone removed by the experienced surgeons than by the trainees. Conversely, for the less critical steps there would be less difference between these groups.

### Participants

Seven consultant and six trainee ENT surgeons performed the entire preparation procedure of the temporal bone for cochlear implantation on the University of Melbourne VR temporal bone surgery simulator. Ethics approval for this study was granted by the Human Research Ethics Committee of the Royal Victorian Eye and Ear Hospital, East Melbourne, Australia (Approval ID: 12/1075H). Written informed consent was obtained from all participants.

### Derivation of surgical regions

For the analysis of bone removal, the specimen was divided into different anatomical regions. Anatomical structures are used as landmarks by surgeons during a procedure, and as such, this spatial division is a natural way of segmenting a surgical procedure into steps. Further, areas associated with anatomical structures are inherently related to the technique and location of bone removal with the surgical drill.

The virtual specimen in the simulator is represented in the volumetric 3D grid, where a voxel represents a grid cell in 3D space. Each voxel contains an attribute to indicate whether it belongs to an anatomical structure segment or the temporal bone. To divide the specimen into regions, standard morphology operations in image processing (dilation and erosion) were performed on the voxels belonging to anatomical structures [11].

**Fig 2:**
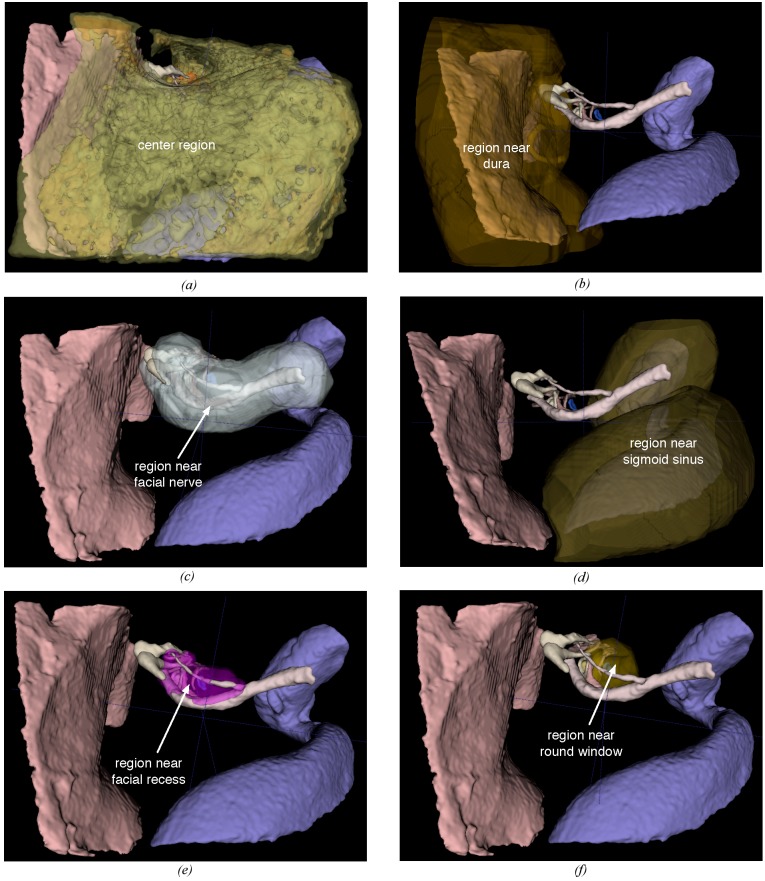
Region divisions in the temporal bone. (a) center region, (b) region around the dura, (c) region around the sigmoid sinus, (d) region near the facial nerve, (e) posterior tympanotomy (facial recess), and (f) region around the round window.

To obtain the regions around structures such as the dura, sigmoid sinus, facial nerve, and round window, the structure boundaries were expanded in each direction in 3D space, using a dilation operation. To obtain areas between structures such as the facial recess, a dilation operation was followed by an erosion (shrinking of the structure boundaries), which is a commonly used operation for filling holes. The width of the area around an anatomical structure to be considered as a region was chosen per structure manually by an expert. The area between the dura and the sigmoid sinus which was not part of the computed drill regions was considered to be the center region where surgeons would make their initial cut into the mastoid bone. The region divisions of a temporal bone specimen are illustrated in Fig 2.

### Determination of variation

To determine drilling variations between expert and trainee ENT surgeons, the number of participants in each group that drilled each voxel in each region was calculated. This measure was normalized to obtain the percentage of participants who drilled each voxel to compensate for unbalanced participant numbers. This percentage value for each voxel in the expert and novice groups was compared using a sign test. The voxels no surgeon drilled were ignored.

To determine the variation within each group, a similarity measure was defined as $\frac{\alpha_{75\%}}{\alpha_{25\%}}$, where *α*_*m*_ refers to the number of voxels drilled by at least *m*% of participants in the group. The thresholds of 25% and 75% were chosen as they are conventional numbers used in descriptive statistics to divide data sets into groups. This measure has two advantages. First, it removes voxels drilled by a low number of surgeons, thus removing any outliers in the data. Second, by considering voxels drilled by a majority of surgeons, but not necessarily all of them, it accounts for small variations that naturally occur when performing a surgical procedure. A high value in the similarity measure represents high coherence in the areas drilled by surgeons within a group in each region.

### Data analysis

The non-parametric sign test was used in the comparison of groups to avoid assumptions of normality. A confidence interval of 95% was considered when testing for significance (*p* ≤ 0.05). Data analysis was performed using Matlab R2015a.

## Results

**Fig 3:**
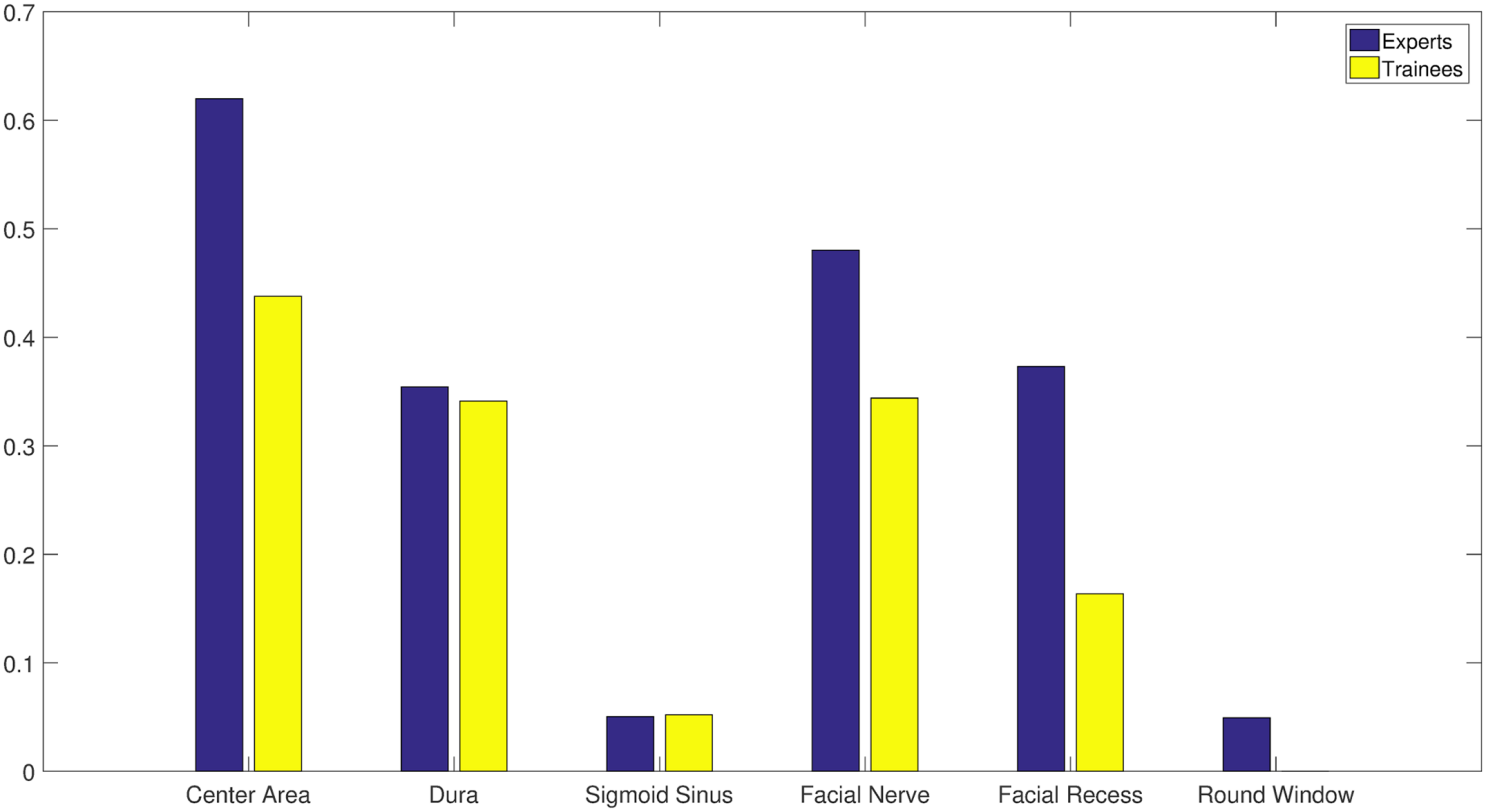
Similarity of drilled areas in different regions of two groups. Similarity is defined as the ratio of the number of voxels in a region drilled by at least 75% of the surgeons in a group and that drilled by at least 25% of the surgeons.

Results of the sign tests indicated that the expert and novice groups showed significant differences when drilling in the regions of facial nerve (p<0.001), facial recess (p<0.001), and round window membrane (p<0.001). In these areas, the similarity of the drilled areas, within the expert group was higher (see Fig 3).

**Fig 4:**
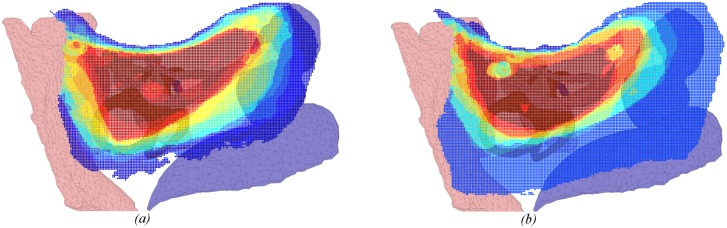
Heat map of drilled voxels in the central area for: (a) expert and (b) trainee groups. No significant difference was observed between groups.

**Fig 5:**
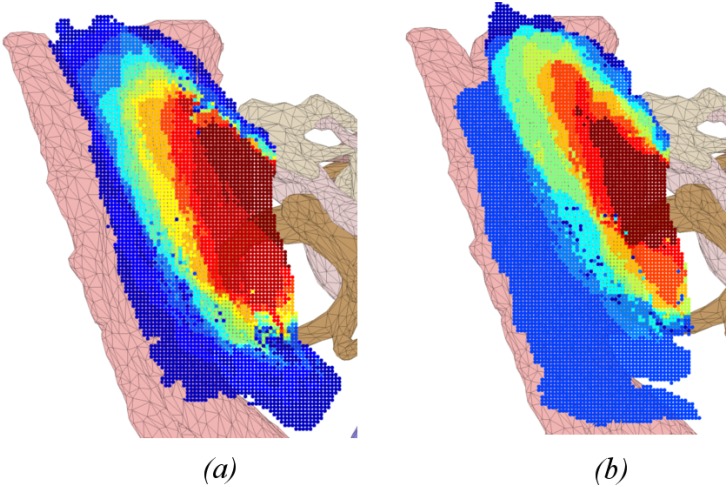
Heat map of drilled voxels around the dura for: (a) expert and (b) trainee groups. No significant difference was observed between groups.

**Fig 6:**
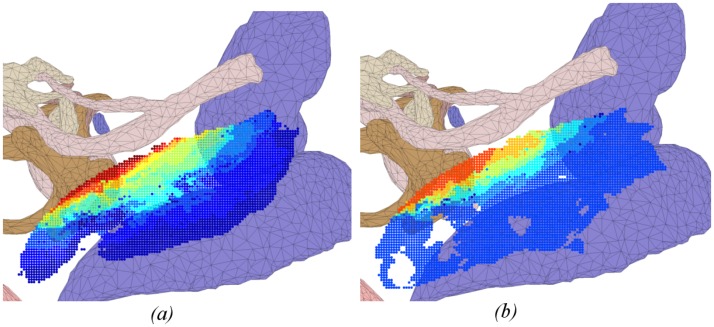
Heat map of drilled voxels around the sigmoid sinus for: (a) expert and (b) trainee groups. No significant difference was observed between groups.

**Fig 7:**
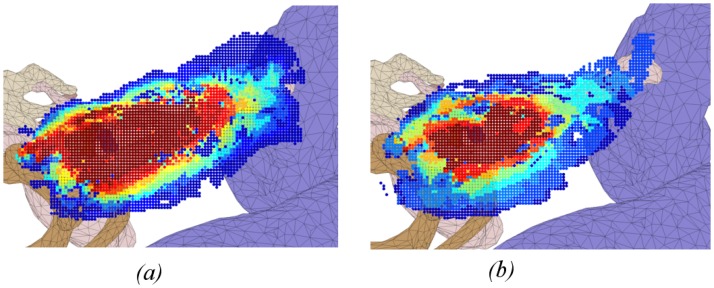
Heat map of drilled voxels near the facial nerve for: (a) expert and (b) trainee groups. Significant differences were observed between groups. Experts were more similar in their drilling in this region than the trainees.

**Fig 8:**
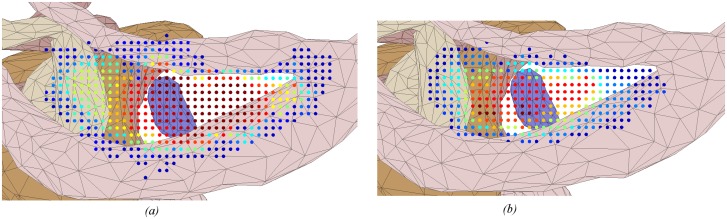
Heat map of drilled voxel in the facial recess for: (a) expert and (b) trainee groups. A significant difference was observed between groups. The expert group was more similar in how they drilled in this region compared to the trainees.

**Fig 9:**
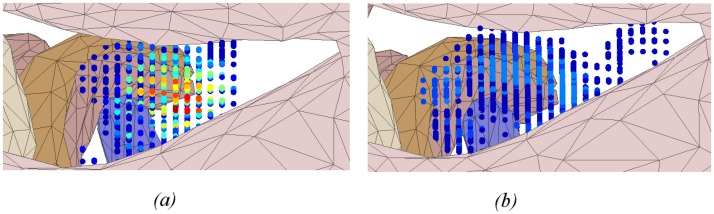
Heat map of drilled voxel around the round window membrane for: (a) expert and (b) trainee groups. A significant difference was observed between groups. The expert group was more similar in how they drilled in this region compared to the trainees.

Heat maps in Figs 4–9 illustrate the differences in drilled areas of the two groups in each region. At each voxel, the colour variation indicates the percentage of surgeons that drilled that voxel. The colour changes from dark blue through light blue, yellow, orange, and red to dark red indicating a gradual increase in percentage from lowest to highest.

## Discussion

We have found that experts exhibited greater concordance in the bone drilled during the critical stages of the procedure (skeletonising the facial nerve, drilling in the facial recess, and exposing the round window), than the less-critical cortical mastoidectomy stage (central region and areas around the dura and sigmoid sinus). There was no significant difference in the consistency of bone drilled by experts and residents in the cortical region, while experts had significantly greater consistency than residents in the critical stages of the procedure. These findings support the hypothesis that experts adopt a consistent approach at critical stages during an operation, but at other times are more flexible.

The heat maps for the regions of the facial nerve (Fig 7), facial recess (Fig 8), and round window (Fig 9) show the bone drilled during the critical parts of the operation. Almost all experts opened up the facial recess wide enough for the round window to be viewed completely. On the other hand, most of the trainees did not drill enough of the posterior tympanotomy and thereby encountered difficulties when exposing the round window. Visualisation of the round window was achieved by experts exposing the facial nerve and chorda tympani thoroughly, and more completely than trainees. In accordance with expert advice on performing a cochleostomy [12], visualization of the round window is required in order for surgeons to have sufficiently good landmarks to choose the correct location of cochleostomy. We find that experts do this in a consistent manner with little variation, both through their preparation of the posterior tympanotomy and also the attention given to removing the bone overlying the round window.

While the surgery of trainee surgeons varied much more than that of experts during the critical stages of the surgery, their drilling behavior was quite similar to that of experienced surgeons during earlier stages of the surgery. The cortical mastoidectomy in this particular operation is simply an approach to find the facial nerve and perform the posterior tympanotomy, which means that full exposure of all of the anatomical structures encountered was not necessary. It appears from these results that surgical residents have the knowledge and technical skill to perform the cortical mastoidectomy adequately. There could be several explanations for the lack of consistency during the critical phases of the operation. It may be tarinee surgeons did not have the knowledge to appreciate the need for a consistent approach, or it may be that they lacked the surgical skill to execute one. Differentiation between these factors may prove to be difficult. Trainees knew where to find the facial nerve and chorda tympani, but did not remove bone overlying these nerves adequately. These trainees were likely apprehensive, as they would have been aware that permanent impairment results from injuring the nerves. In addition, considerable technical skill is required in order to skeletonize them. Finally, trainees need to appreciate that skeletonization is necessary in order to visualize the round window. The extent to which the relative inexperience of a trainee surgeon manifests in fear, more limited technical ability or less extensive knowledge is difficult to ascertain.

These observations are consistent with the broad literature on models of skills development [13], expertise [14, 15] and the expert-performance approach [16]. Eraut [7, 17] states that experts no longer rely on rules or guidelines; have an intuitive grasp of situations based on a deep tacit understanding; employ analytic approaches used only in novel situations or where problems occur; and have a vision of what is possible. According to Dreyfus et al. [14, 18], the expert, having progressed through novice, competence and proficiency stages has high levels of procedural knowledge and skills (knowing how) as well as declarative knowledge (knowing what), and contextual flexibility (knowing when and where). Dunphy and Williamson [5] note that expert performance does not rely on principles or rules to connect understanding of the situation to an appropriate action.

The variations observed in the non-critical aspects of the procedure show that the experts in our study were comfortable in their practice to take shortcuts and develop their own style of surgery because they possessed the intuition and tacit knowledge to do so safely. Indeed, knowing when to follow rules, and when not to, in itself is an aspect of expertise embodied in the characteristics of “pattern recognition”–the ability to identify complex patterns, and “flexibility”–the ability to approach each situation with a high level of flexibility [5]. Moulton et al. describe this approach as “slowing down when you should” to stay out of trouble during surgical practice [19]. It is not necessarily a slowing down temporally but a shift from automaticity to effortful modes. Indeed a subset of surgeons involved in their qualitative studies “vehemently objected to ever being in the automatic mode”. Others acknowledged the state of “drifting” describing approaches to routine practice in which they may not have engaged in the continuous essential monitoring of their progress. What is important here is that effortful modes require cognitive space to deal with the changing conditions of the procedure. From this perspective, the experts in our study are likely to have adopted this effortful mode as they approached the critical points of the procedure.

The strength of this study is that it enables us to directly compare expert and trainee surgeons’ performance of critical and other aspects of surgical preparation, with cochlear implantation on a virtual temporal bone as the “test case”. This study offers insight to the level of skills development–*product-oriented* (the end result) rather than *process-oriented* (how things are done). Given the significant differences between trainees and experts, this VR environment offers tremendous research opportunities to focus attention on the process-orientation to gain deeper understanding of how the expert is achieving superior performance. This is a reflection of the type of information that can be gleaned with such a system—a continuous stream of data on how a surgeon handles instruments and interacts with the anatomy that they encounter. However, this strength also carries a limitation; the skills are performed out of context and therefore may not reflect the environmental and sociocultural pressures of real clinical environments (i.e. the operating theatre) in which trainee and expert surgeons are expected to perform [20, 21]. In some ways then, these results are even more striking since trainees are likely to be under greater pressure than experts in real work settings. It is also important to keep in mind that the models of skills development and expertise we have outlined focus on individuals and that safe surgical operating extends beyond the expertise of one individual.

The critical points in this surgical simulation may be better represented by a different theoretical approach. *Threshold concepts* are described as being “akin to a portal, opening up a new and previously inaccessible way of thinking about something. They represent a transformed way of understanding, interpreting, or viewing something without which the learner cannot progress” [22]. Threshold concepts are further characterized as irreversible, in that once acquired the change in perspective or behavior which has been produced by the threshold concept is unlikely to be unlearned, it may even be difficult to comprehend the state of those on the *other side* of the threshold who are unable to comprehend the meaning of what is happening because they do not understand it [22]. Threshold concepts are transformative in that they lead to a change in perception and in certain powerful instances, this change may have an associated shift in identity. We posit that the critical points in this simulated surgical environment represent a threshold concept. Trainee surgeons are in a *liminal* state where they may recognize the critical points but are not yet able to successfully progress to the expert state because they have not undergone the required ontological shift associated with being an expert surgeon. Although mimicry of superior performance will likely add value in VR, true expertise demands the ability to deal with contextual complexity currently not represented in our simulation.

These results provide valuable insights into expert behavior. Surgeons do act similarly when the stakes are high in an operation—they revert to standardized procedure, even though exhibiting flexibility at other times. In other words, it appears that experienced surgeons adopt the view that surgical error is least well tolerated when the consequence or a mistake is greatest, and that the best way to mitigate this risk is to adopt a standard approach at these critical moments during surgery, what Moulton et al describe as moving to an “effortful mode”. This is akin to the “standard operating procedures” (SOP) adopted in aviation or other contexts when risk is high and failure catastrophic. This supports the practice of surgeons “following” the leadership of esteemed colleagues, and emphasizes the value of following prescriptive descriptions of complex surgical cases.

## Supporting information

S1 File This file contains the data and Matlab code used in the discussed study. Extract the zip file and run the file ‘main.m’. The folder ‘Data’ holds the anonymized expert and trainee data.
(ZIP)
